# Climate change and *Ixodes* tick-borne diseases of humans

**DOI:** 10.1098/rstb.2014.0051

**Published:** 2015-04-05

**Authors:** Richard S. Ostfeld, Jesse L. Brunner

**Affiliations:** 1 Cary Institute of Ecosystem Studies, PO Box AB, Millbrook, NY 12545, USA; 2 School of Biological Sciences, Washington State University, Pullman, WA 99164, USA

**Keywords:** climate warming, global climate change, disease ecology, tick-borne disease, Lyme disease

## Abstract

The evidence that climate warming is changing the distribution of *Ixodes* ticks and the pathogens they transmit is reviewed and evaluated. The primary approaches are either phenomenological, which typically assume that climate alone limits current and future distributions, or mechanistic, asking which tick-demographic parameters are affected by specific abiotic conditions. Both approaches have promise but are severely limited when applied separately. For instance, phenomenological approaches (e.g. climate envelope models) often select abiotic variables arbitrarily and produce results that can be hard to interpret biologically. On the other hand, although laboratory studies demonstrate strict temperature and humidity thresholds for tick survival, these limits rarely apply to field situations. Similarly, no studies address the influence of abiotic conditions on more than a few life stages, transitions or demographic processes, preventing comprehensive assessments. Nevertheless, despite their divergent approaches, both mechanistic and phenomenological models suggest dramatic range expansions of *Ixodes* ticks and tick-borne disease as the climate warms. The predicted distributions, however, vary strongly with the models' assumptions, which are rarely tested against reasonable alternatives. These inconsistencies, limited data about key tick-demographic and climatic processes and only limited incorporation of non-climatic processes have weakened the application of this rich area of research to public health policy or actions. We urge further investigation of the influence of climate on vertebrate hosts and tick-borne pathogen dynamics. In addition, testing model assumptions and mechanisms in a range of natural contexts and comparing their relative importance as competing models in a rigorous statistical framework will significantly advance our understanding of how climate change will alter the distribution, dynamics and risk of tick-borne disease.

## 1. Introduction

Anthropogenic climate change over the past century has led to mean global temperatures consistently above those historically recorded as well as to increased variability in temperature and precipitation [1]. One of the most discussed, but perhaps least understood consequences of global climate change is its effect on infectious disease and human health [2,3]. Although there is no shortage of predictions for how infectious disease will expand or shift in range, most are built upon the assumption that current climatic conditions are what limit the distribution of these diseases, e.g. [4]. Whether or not this assumption holds, however, is rarely known [5,6], in part because of difficulties demonstrating the cause(s) of absence [7]. This is particularly true for tick-borne diseases where data on mechanistic relationships between climate and physiology of the tick vector and pathogen are often limited.

Climate change is frequently invoked as a primary cause of expansions in incidence of tick-borne diseases, particularly Lyme disease. In the nearly 40 years since Lyme disease was first described, both the number and geographical range of reported human cases have increased dramatically in North America [8–10] and Eurasia [11,12]. Other tick-borne diseases, including tick-borne encephalitis, human babesiosis and granulocytic anaplasmosis, have also emerged and expanded on these continents. Although these increases in incidence and range have been occurring simultaneously with warming trends, the effects of climate are difficult to disentangle from other potential causes. First, case reports typically increase with increasing awareness of emerging diseases on the part of healthcare providers and the public, but these trends might not reflect actual increases in infection or disease. Second, ticks and tick-borne diseases may be expanding for reasons that have nothing to do with climate, for instance, because of recolonization of regions that were previously unsuitable following large-scale land clearing, but with no clear impact of climate change [13]. Thus, even when climate change, tick population dynamics and epidemiological patterns are correlated, causation is difficult to establish and specific causal factors hard to confirm.

An additional challenge in understanding the impacts of climate change is the sheer number of components in the biology of vectors, hosts and pathogens that are potentially influenced by climate, including survival rates and phenology of immature and mature ticks, fecundity, host encounter rates, vector competency, as well as replication rates of the pathogen and the community dynamics of hosts. The myriad climatic variables, influences and interactions have posed a significant challenge to researchers attempting to understand and predict the net effect of climatic conditions and climate change on tick-borne disease [14]. Indeed, it is likely that climate change will have countervailing influences on different components of the disease system. Warmer conditions, for instance, often not only lead to faster rates of tick development [15] but also to reduced host-seeking activity [16] and stronger immune responses in some vector taxa [17].

Despite these challenges, the importance of understanding and predicting the consequences of climate change for the distributions and intensities of ticks and tick-borne infections is only increasing as the global climate continues to warm. This is particularly true for *Ixodes* ticks and the pathogens they transmit, which are the focus of this review. Two main approaches have been used to project how the distribution of *Ixodes* and the pathogens they transmit will change as the climate changes. The first approach consists of an effort to find the set of environmental factors that best explains the current distribution of ticks (or of tick-borne disease) and, assuming that those conditions represent suitable habitat, project future distributions using models that estimate where that set of conditions will occur in the future. This approach is largely *phenomenological*, in that environmental factors are selected based on correlations between those factors and the presence (or less commonly, the abundance) of ticks or of tick-borne disease, but typically without knowledge of underlying mechanisms. The second attempts to determine the relationship between specific environmental factors and at least some tick-demographic transitions, and asks which geographical locations currently or in the future provide the environmental conditions that allow tick populations and their associated pathogens to persist or grow. This approach is largely *mechanistic*, in that it focuses on the mechanisms by which abiotic factors affect tick-demographic parameters. Importantly, both approaches also assume that abiotic conditions are predominantly important, an assumption we return to in §2.

Both approaches offer insight but have serious limitations [18–20]. The phenomenological approach generally employs spatially or temporally broad and ecologically relevant datasets, but risks basing models on spurious rather than causal relationships when it does not first establish the mechanisms governing the effects of environmental variables on tick demography. In addition, the absence or rarity of ticks in certain regions is often assumed to be caused by unsuitable (current) conditions in that region, but such unsuitability is rarely if ever established [21,22]. Lastly, the different spatial and temporal scales of these phenomenological studies, not to mention the different climatic variables they include, have made it difficult to identify and evaluate consistent patterns in associations [23]. The mechanistic approach, on the other hand, typically uses controlled experiments to determine the effects of specific environmental conditions on a handful of specific developmental or demographic responses. Because the environmental conditions are usually imposed in laboratory settings, which necessarily exclude many dimensions of ecological realism (e.g. variability in conditions, interacting species), one must take care in extrapolating relationships observed in the laboratory to the field. Field experiments can provide more ecologically relevant data but are usually of limited scope focusing, for instance, on only a subset of relevant demographic processes, often in few locations, or physically restraining ticks in a way that complicates interpretation of survival patterns [24–28]. The advantage of this approach is that projections are based on mechanistic relationships rather than statistical associations. But while the models used to integrate these empirical studies have become increasingly sophisticated [29–32], they are still based on few empirical data and best guesses about key parameters.

Recent advances in modelling effects of climate change on both free-living and parasitic species offer promising avenues for assessing climate effects on tick-borne disease. For instance, hierarchical analyses of both the fundamental and realized niches of the key interacting species (e.g. parasite, vector, reservoir host, ‘accidental’ host), at multiple spatial scales can help bridge the gap between phenomenological and mechanistic approaches [33]. Similarly, Liu *et al.* [34] provide an example of a species distribution model applied to a pathogen in which habitat structure, host diversity and propagule pressure are considered, while autocorrelation and sampling bias are statistically controlled. Also, the recent application of next-generation matrix models to tick-borne diseases has provided a robust means of identifying the most important demographic and epidemiological processes in the establishment of ticks and tick-borne disease.

In this paper, we review the literature on: laboratory and field studies linking abiotic conditions to tick demography and behaviour (§2); phenomenological models of ticks and tick-borne diseases affected by climate (§3); mechanistic models of tick dynamics and distributions as affected by climate (§§4 & 5); climate effects on important hosts and pathogens (e.g. via changing phenologies) (§§6 & 7). Last, in §8, we will point out knowledge gaps and suggest new approaches.

## 2. Laboratory and field studies linking abiotic conditions to tick demography and behaviour

**Figure 1.**
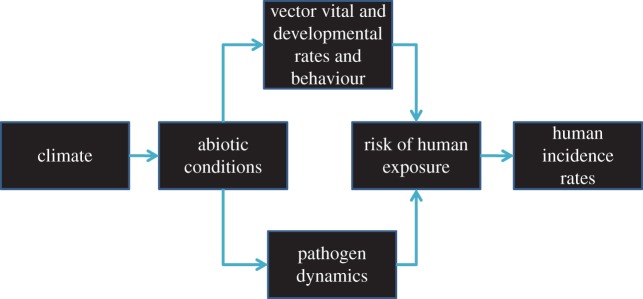
Conceptual model linking climate change to tick-borne disease in humans. Each arrow linking boxes represents an opportunity to fill in knowledge gaps. For example, determining how climate change at global and regional scales influences the key abiotic variables for ticks at local scales is critical. A mechanistic understanding of how specific abiotic variables interactively determine demographic, developmental and behavioural processes in tick vectors and dynamics of tick-borne pathogens requires further development. How climate-driven dynamics of ticks and pathogens affect risk of human exposure, how non-climatic factors influence risk, and how risk is translated into disease incidence are crucial variables for further study. (Online version in colour.)

Most *Ixodes* ticks spend more than 95% of their lives on or just below the ground surface digesting a blood meal, moulting, in diapause or seeking a host (figure 1). The conditions at the ground thus seem critically important to tick survival and habitat suitability. Research on * Ixodes scapularis* conducted around the time of the discovery of Lyme disease, indicated that tick populations in coastal Massachusetts, USA, were only found after mild winters [35,36]. At this time, *I. scapularis* in the northeastern United States appeared to be restricted to coastal southern New England [13,37], a zone of relatively cool summers and warm winters compared with inland sites. These observations led to the conclusion that extreme cold and heat were unsuitable for this species, limiting its distribution and affecting its population dynamics. At the time of these observations, researchers mistakenly considered their study organism a distinct, northern species of tick that had been named *I. dammini*. Only later, when it was revealed that the New England ticks under study belonged to populations of a widespread species, *I. scapularis*, were many of the conclusions revised [14]. The presence of *I. scapularis* in much colder climates of Minnesota and Wisconsin and in much warmer climates of Georgia and South Carolina provided evidence that the species tolerated a considerably wider range of conditions.

Later laboratory experiments repeatedly demonstrated the importance of humidity and temperature for tick survival [15,38–40]. *Ixodes scapularis* is highly susceptible to desiccation when relative humidity drops below approximately 90% [38]. Even a few hours at low humidity can be lethal, but ticks returned to humid air may suffer little mortality [40]. As ticks are very capable of avoiding desiccating conditions, for instance by remaining in the humid leaf litter or soil until conditions become more favourable, dry conditions may not directly restrict tick populations, although long dry periods can reduce longevity [41]. Environmental conditions may also constrain ticks' ability to quest for hosts. In summer, for instance, high temperatures and low relative humidity limit tick movement and questing activity [42]. Thus, hot and dry summer conditions may delay or prevent ticks from obtaining the necessary blood meal. This delay may not be very restrictive as questing ticks seem able to survive for several months or longer [24–26,43]. However, there may be combinations of biotic and abiotic conditions where ticks rarely quest and encounter blood-meal hosts, which could potentially limit tick populations.

Comprehensive experiments with *I. ricinus* similarly illustrate the interactive effects of temperature and humidity on survival of questing immature stages [44–48]. Low relative humidity (*ca* 50%) permits survival when temperatures are below 5°C, but relative humidity above *ca* 70% is required when temperatures exceed *ca* 15°C. These studies also have shown that *I. ricinus* employs sophisticated movement patterns to seek favourable microclimatic conditions, e.g. under leaf litter or in the soil, when air becomes too warm or dry. For instance, a large saturation deficit reduces activity levels of *I. ricinus* but apparently has little impact on survival because it is unrelated to tick abundance in the subsequent year [49]. As a consequence of these behavioural adaptations, the relevance of laboratory-determined lethal conditions to demographic patterns in nature remains elusive. Climatic restrictions on questing behaviour and host encounters (e.g. saturation deficits; [50]) deserve more research.

Cumulative temperature appears to control the length of development of most developmental stages of *I. scapularis*. The ability of ticks to oviposit and eclose within a single season depends on cumulative degree days, although the precise number of degree days varies between studies (e.g. [24,51]). Similarly, rates of development of immature ticks in the field and laboratory are correlated with temperature [15,52]. These results have led several authors to postulate that high mortality associated with longer developmental periods might limit the distribution of *I. scapularis* to warmer regions, but that climate warming will cause northward range expansion [15,31,32,53–55].

Cold winter temperatures appear to have a more direct effect than do warm temperatures on *Ixodes* survival and population dynamics. Newly moulted nymphal and gravid female *I. scapularis* overwinter before emerging in spring to quest for a blood-meal host or oviposit, respectively. Even short-term exposure to extreme cold (e.g. below −15°C) can be lethal to *I. scapularis* [39,56] and *I. ricinus* [44] so cold winter weather could be responsible for a significant amount of mortality, and might constrain *Ixodes* populations. Establishing the lower critical temperatures in the laboratory, however, is similarly of dubious relevance to climatic limitations in nature, if ticks can escape extreme conditions for sufficient periods. A recent field experiment by Brunner *et al.* [57], for instance, found that the hazard of overwintering mortality of nymphs in field enclosures was essentially constant in two locations, regardless of temperature. In addition, a recent laboratory study by Herrmann & Gern [58] suggested that frequent temperature variation may be more important for *I. ricinus* survival than low temperatures, so relating laboratory results to natural environments remains a challenge.

Nevertheless, increasingly strong evidence indicates that the latitudinal and altitudinal limits of *I. ricinus* and *I. persulcatus* distributions in Europe are determined by cold temperatures. Expansions of tick populations northward and to higher elevations correlated with climate warming have been documented in Sweden [59], Norway [60,61], European Russia [62], the Czech Republic [63] and the UK [64]. Tick density and/or prevalence of infection with tick-borne pathogens such as tick-borne encephalitis virus (TBEV) is correlated with either temperature (positively) or altitude (negatively) in Switzerland [65], Norway [61] and Sweden [66,67]. And similarly, incidence of tick-borne disease correlates with mild winters and warm, humid summers in Sweden [67,68], Hungary [69], Slovakia [70] and European Russia [62]. Importantly, climatic effects on human behaviour that could influence their contact rates with ticks, independently of climatic effects on ticks, also might contribute to these correlations [66].

**Figure 2.**
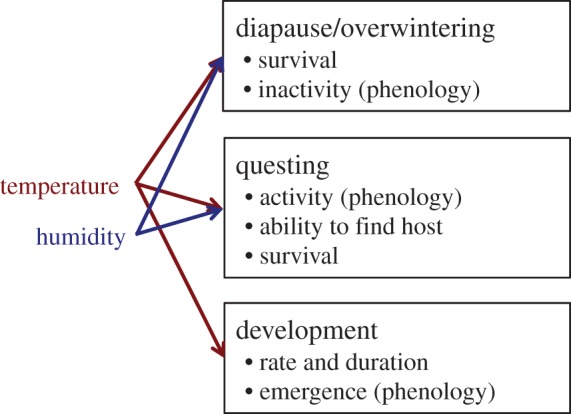
Key processes in the life cycle of ixodid ticks controlling their population dynamics and effects on risk of exposure to tick-borne pathogens. Arrows indicate the potential for temperature and humidity variables to affect each process. (Online version in colour.)

In summary, while there are good reasons to think that the survival and development of *Ixodes* ticks may be limited by temperature and humidity (figure 2), these limitations may only manifest at the extremes of their ranges and where ticks are not able behaviourally to avoid inhospitable conditions. Within regions that have more permissive conditions, the direct effects of temperature and humidity may be more subtle or even relatively unimportant compared, for instance, with the abundance of hosts (e.g. [71]).

## 3. Phenomenological models of ticks and tick-borne disease

Given these potential environmental controls on ticks and tick-borne disease, there have been many efforts to find the set of environmental factors that best explains the current distribution of ticks (or of tick-borne disease). Then, assuming that those conditions represent the full range of suitable habitat, projections of this set of conditions in the future are used to estimate the future distributions of ticks or disease. This approach is largely phenomenological, in that environmental factors are selected based on correlations between those factors and the presence (or less commonly, the abundance) of ticks or of tick-borne disease. In an important early example of this approach Brownstein *et al.* [72] modelled habitat suitability for *I. scapularis* in the USA. The authors used a tick distribution map [73] to discriminate locations with from those without *I. scapularis*. They used 16 climatic variables in a remote sensing dataset—the mean, minimum, maximum and standard deviation of four monthly ground temperatures—to create a climate envelope of suitability. The resulting habitat suitability map predicted modest westward expansion but little northern expansion of tick populations with climate change. In marked contrast, a climate envelope model of Lyme disease cases by Ashley & Meentemeyer [74], based only on spring temperature, precipitation and soil moisture, predicted that vast areas of the eastern and midwestern United States outside the current range were suitable for invasion by Lyme disease. As is often the case with such phenomenological models, however, the important predictor variables can be chosen arbitrarily and are often hard to interpret biologically. For example, the potential for winter, autumn and summer variables to delimit ticks and tick-borne disease was not considered by Ashley & Meentemeyer [74]. In the Brownstein *et al.* [72] study, the best predictor of tick habitat suitability was minimum winter temperature, but the relationship was a fourth-order polynomial that has no obvious biological interpretation. It is also far from clear if such statistical associations can be expected to hold in the future or even new datasets. Only recently have climatic variables been considered together with factors like habitat fragmentation and host abundances [34,75,76].

For *I. ricinus* in Europe, Estrada-Pena [77,78] used remotely sensed data on mean monthly temperature and normalized difference vegetation index (NDVI; an index of plant photosynthetic activity reflecting combined moisture and temperature) to generate a climate suitability envelope. This model was then used to hindcast changes in the distribution of suitable habitat between 2000 and 2010, showing spreading suitable conditions that roughly match apparent increases in tick distribution. Estrada-Pena *et al.* [79] suggest that continued climate warming will cause a continued range expansion for this tick and potentially the pathogens it transmits, reinforcing the predictions based on process models described in §§3 & 4. Similarly, Porretta *et al.* [80] developed a MAXENT climate envelope model from over 4500 tick occurrence locations using 19 bioclimatic variables derived from monthly rainfall and temperature data. Their model predicted a massive spreading of habitat suitability throughout northern Europe and northwestern and northcentral Russia by 2050. Both the more severe greenhouse gas emission scenario (A2) and more modest (B2) scenario produced similar predicted spread, although the B2 scenario generally predicted greater habitat suitability within the range [80].

As pointed out by Estrada-Pena *et al.* [79], and Porretta *et al.* [80], such bioclimatic modelling results must be considered provisional because they incorporate only some of the variables known to affect ticks and tick-borne diseases. Without first establishing the mechanisms governing the effects of environmental variables on tick demography, the phenomenological approach risks basing models on spurious rather than causal relationships. In addition, the absence or rarity of ticks in certain regions could be caused by insufficient time for ticks to disperse from the established range rather than by unsuitable current conditions [21,22]. In general, for species like *I. scapularis* and *I. ricinus* that are rapidly expanding their geographical range, the environmental conditions correlated with their distribution at any given time are necessarily a subset of all permissible conditions and thus must underestimate the range of conditions under which they can exist.

If temporal trends in Lyme disease incidence are correlated with temporal trends in climate, this would suggest that long-term climate change will cause directional changes in the public health burden of tick-borne disease. An early study of correlations between climate variables and Lyme disease cases was performed by Subak [81], who collated the annual number of Lyme disease cases between 1993 and 2001 in seven states from the northeastern United States. Subak asked whether the Palmer Hydrological Drought Index (PHDI, a measure of summer moisture) and the average winter temperatures in these states predicted Lyme disease incidence the following year or 2 years later. Although winter temperatures were poor predictors, PHDI 2 years previously correlated positively with Lyme disease incidence for four of the states, although a plausible mechanism was not provided [81]. Analysing the same dataset, McCabe & Bunnell [82] concluded that Lyme disease cases in the northeastern United States were positively correlated with precipitation during May and June of the same year, but not with precipitation in any prior years. Perhaps because of these conflicting conclusions and the lack of a mechanistic understanding of processes that might underlie the correlations, not to mention the issues with identifying limiting conditions for a process that is generally increasing [83], researchers generally turned to constructing mechanistic models of tick population dynamics and climate envelope models of habitat suitability for the tick vectors.

## 4. Mechanistic models of tick abundance, population dynamics, phenology and distributions

While mechanistic models have the advantage of being able to project biological mechanisms, as opposed to associations, forward in time, the sheer numbers of both climatic parameters and tick-demographic and behavioural response variables pose enormous challenges to the constructing and understanding mechanistic models. Most mechanistic models, therefore, embody one or a few relationships that are thought to be dominant. The approach by Ogden *et al.* [53], using the average number of degree days greater than 0°C to describe present and project future habitat suitability with climate warming, has been very influential (e.g. [84]). The authors built their model around the relationships between the key developmental rates (e.g. moulting, eclosion) and temperatures that they had observed in the field for *I. scapularis* near the northern extent of their North American distribution [26]. Because of data limitations they tuned many parameter values, including those determining density-dependence and on-host mortality, to reproduce observed patterns. Their simulation model, which included 48 parameters for tick vital rates and two parameters for host-finding, was reasonably successful in mimicking natural patterns of population dynamics for all three blood-feeding stages [53]. Not surprisingly given its underlying assumptions, this model and several follow-up studies [32,54,85,86] suggested that the average number of degree days greater than 0°C was the key climatic parameter limiting tick distribution; that vast areas outside the current distribution of *I. scapularis* were suitable and that the tick's geographical range would expand northward and westward well into Canada as the climate warms. However, the model results rest heavily on the assumption that off-host tick mortality rate is a time-constant variable, such that the number of ticks surviving decays exponentially with time in each developmental stage, a reasonable first approximation, but an important assumption that needs testing.

More recent models of the influence of climate on *I. scapularis* in North America and *I. ricinus* populations in Europe still embody the temperature-dependent development of ticks at their core [30–32,87], but have begun to use matrix-modelling approaches, which offer the potential for more robust sensitivity analyses [32]. Such sensitivity or elasticity analyses can help identify the parameters that most strongly influence tick population dynamics and are thus the most important to validate. However, these models, like any other, simply expose the consequences of the assumptions that they make [88]. Wu *et al.* [32], for instance, essentially recreated the model of Ogden *et al.* [53] using a set of periodic differential equations. It is not surprising that they found that variation in summer temperatures and the specific temperature-dependent rates of development were key parameters. We are not aware of any study with mechanistic models that explicitly considers alternate assumptions or hypotheses (e.g. as alternative model formulations). While these complex mechanistic models can reproduce the general patterns of tick population dynamics, it is unclear which biological mechanisms are necessary and sufficient to do so, and which simply add degrees of freedom. In other words, these models might often make the ‘right’ predictions for the wrong reasons. We see the overparameterization of models as an important deficiency that needs to be addressed and encourage researchers to consider multiple competing hypotheses in their models.

In a related vein, these complex models have rarely been rigorously statistically tested against empirical data. While these models can produce many response variables (e.g. the presence, peak or overall density and phenology of tick populations), model output does not necessarily match the available data (e.g. [89,90]). Indeed, finding *any* data with which to test models properly has sometimes required a great deal of creativity [85]. One common approach has been to compare the predicted and observed phenologies by gestalt [53], correlations [30] or ad hoc methods [85], but what constitutes an adequate or good fit is subjective. Models are rarely challenged with independent datasets from other regions or times. Dobson *et al.* [30] took the commendable step of challenging their model with data from two independent sites and found substantial deviations between their model's predictions and observed dynamics at these sites. Determining which parameter values needed to change to improve the model fit in each location proved enlightening [30], but we again caution that with so many parameters to tweak, these insights should be viewed as hypotheses rather than fundamental lessons about tick biology. Even with data limitations, we suspect a great deal of rigour and clarity may be gained by incorporating model fitting and validation into formal statistical (i.e. likelihood or Bayesian) frameworks.

Lastly, all these models continue to rely on few empirical data and best guesses about key parameters. For example, the effects of changing photoperiod and temperature on tick diapause and the dependence of tick feeding probability on host density and tick density are poorly known yet critical parameters affecting model results [30–32,87]. The usefulness of these models, therefore, is likely to increase when field data linking specific abiotic variables to specific demographic and behavioural responses are available.

It is noteworthy that despite their divergent approaches, both mechanistic and phenomenological models suggest dramatic range expansions of ticks and tick-borne disease as the climate warms. However, each modelling exercise has produced different maps based on different assumptions about the impacts of abiotic conditions on ticks and tick-borne pathogens. These inconsistencies, serious data limitations regarding the key tick-demographic and climatic processes, the limited ability to demonstrate situations in which climatic conditions are responsible for prohibiting tick-borne disease, and only limited incorporation of non-climatic processes, have weakened the application of this rich area of research to public health policy or actions.

## 5. Models of *R*_0_

The recent application of next-generation matrix (NGM) models to tick-borne diseases has provided some useful guidance about which demographic and epidemiological parameters are most important for the basic reproductive rate of the infection, *R*_0_ [91–96]. *R*_0_, defined as the number of secondary cases caused by a single infected individual in a wholly susceptible population, is particularly useful when considering whether tick-borne diseases can successfully invade and spread. The NGM approach offers a straightforward means of evaluating the sensitivity or elasticity of *R*_0_ to changes in each of a matrix of transition probabilities representing paths through which hosts or ticks become infected. These models consistently indicate that survival from fed larva to feeding nymph is a critical for *R*_0_ for tick-borne encephalitis and Lyme borreliosis [91,95]. This observation is relevant to the spread of tick-borne disease in a changing climate since all of the steps in this transition, from moulting to overwintering survival to survival while questing, are sensitive to climate conditions. Also, when the phenology of ticks and the duration of infectiousness are included in the NGM of Lyme disease in North America, the probability that a nymph finds a competent host is also strongly influential [95]. We suggest that a focus on those parameters with high elasticities will prove most fruitful, especially when considering alternative assumptions and hypotheses in this framework. For instance, most NGM models de-emphasize the importance of tick and host densities (but see [32]), but this is probably because they assume that tick–host encounters are essentially constant [91,95]. Different function forms of host encounter term in epidemic models strongly alter the predictions of these models [97,98].

## 6. Climate effects on hosts

Not only can climate change affect habitat suitability and demography of ticks, it might also affect important hosts necessary for persistence of both ticks and tick-borne pathogens. Several of the models described in §3 & 4 incorporate hosts implicitly by allowing climatic conditions to affect host-seeking activity and presumably host encounter rates (e.g. [15,30,87]); given that a tick is questing, contact rates with hosts are generally assumed to be constant. However, the relevant parameters concerning host-seeking activity by ticks and impacts of climate change on the distribution and abundance of tick-hosts have rarely been incorporated into projections of the future distributions of disease risk. Because the most important hosts for *Ixodes* ticks in areas impacted by Lyme disease and other tick-borne diseases are endotherms, the effect of climate warming is expected to be buffered by physiological temperature regulation. Nevertheless, a recent study by Roy-Dufresne *et al.* [99] indicates that the geographical range of the white-footed mouse (*Peromyscus leucopus*) has extended northward in Quebec coincident with climate warming between 1975 and the present. Roy-Dufresne *et al.* [99] project that, because this mouse is so important both to tick survival [100] and transmission of *Borrelia burgdorferi* [101], *A. phagocytophilum* [102] and *Babesia microti* [103], continued range expansion will facilitate poleward expansion of Lyme disease. Population genetic analyses of white-footed mice in Quebec suggests that, with the exception of large rivers, there are few geographical barriers to gene flow that might inhibit the ability of this species to track a warming climate and expand northward [104]. While some models of tick range expansion in Canada indicate that long-distance dispersal on migratory birds will help ticks and *B. burgdorferi* track suitable conditions for tick survival as the climate warms [105], a recent projection of a *B. burgdorferi* risk index suggests that the expansion of the Lyme disease agent will be limited by the dispersal of *P. leucopus* [106]. The degree to which climate change will alter the spatial distributions, population dynamics and physiology (especially immunology) of hosts for ticks is a poorly studied but promising area for future research.

## 7. Climate effects on phenology and pathogens

Direct effects of climate on tick-borne pathogens have been neglected, but the potential exists for climatic conditions experienced by ticks to influence persistence and replication rates of pathogens before, during and after seasonal diapause. Recently, both theoretical and empirical studies have addressed how climatic conditions, specifically the severity of winter cold, affect genotype frequencies of *B. burgdorferi* via influences on the phenology of larval and nymphal ticks [107,108]. In North American Lyme-disease-endemic regions with relatively mild winters, including the northeastern United States, each year's cohort of larval ticks ecloses and begins seeking hosts in August, about two months after the prior generation's nymphal cohort has peaked in May or June. As a result of this time lag between host inoculation by nymphs and pathogen acquisition by larvae, larvae should acquire only those genotypes of *B. burgdorferi* that are able to persist in hosts for several months [107]. The *B. burgdorferi* genotypes known to persist well in white-footed mice tend to be the ones that are particularly invasive in humans [109–111]. In the Midwestern United States, where winter temperatures are lower and the climate is more extreme, *I. scapularis* exhibits a 3-year life cycle with fairly synchronous nymphal and larval emergence, and the more persistent human-invasive genotypes thus tend to be under-represented [107,112]. Consequently, it is plausible that a warming climate will facilitate dominance by human-invasive strains of *B. burgdorferi* in the Midwest, with potentially strong epidemiological consequences [107]. Similarly, if we assume that larval development and emergence will be accelerated relative to nymphs in a warming Northeast (e.g. if nymphal emergence is primary controlled by photoperiod [113,114]), then we would expect less persistent *B. burgdorferi* strains to become more common. An analysis of tick phenology in a warming climate by Levi *et al.* [115], however, indicates that activity peaks of both larval and nymphal *I. scapularis* s have advanced equally over the past 20 years, in which case the persistent strains should remain dominant.

Transmission by *I. ricinus* and *I. persulcatus* ticks of TBEV to wildlife hosts typically results in short-term infection that might not be disseminated beyond the site of the tick bite. As a consequence, the transmission cycle of TBEV appears to rely largely on simultaneous feeding (co-feeding) by infected nymphs and uninfected larvae in close proximity on the host's body [116]. For TBEV to be maintained in an enzootic cycle that can result in zoonotic transmission, it is often assumed that some degree of synchrony in the seasonal questing activity of larval and nymphal ticks is therefore necessary [117], a view supported by the large contribution of co-feeding transmission to *R*_0_ in an NGM model [91,92]. An NGM model of Powassan virus, a close relative of TBEV, that explicitly included the overlap between larval and nymphal feeding, suggests that climatic factors that reduce their synchrony will reduce enzootic transmission, although modest vertical transmission might still maintain the virus populations [93]. Kurtenbach *et al.* [108] suggested that, like other pathogens that cause only short-lived infections in reservoir hosts, TBEV maintenance might be facilitated by milder climates that permit extended host-seeking activity of both larvae and nymphs, which should result in seasonal overlap and co-feeding. It stands to reason that climate changes that reduce temporal overlap between larval and nymphal feeding would reduce the risk of TBE. Long-term studies will be necessary to evaluate this expectation.

## 8. Knowledge gaps and future directions

A unified conceptual model linking climate change to tick-borne disease would be helpful in guiding better understanding (figure 1). Guided by this conceptual model, future research could explore the specific, local abiotic variables affected by regional or global changes in climate; the impact of those abiotic variables on behavioural, developmental and demographic processes of ticks, and on the dynamics of tick-borne pathogens. An understanding of climatic impacts on the vector and pathogen will allow researchers to predict how risk of human exposure to these pathogens will change as the climate continues to change. And, finally, climate-driven changes in risk, together with changes in human behaviour, will allow epidemiological patterns to be forecast.

While it is clear that the developmental rates and timing of *Ixodes* spp. are temperature-dependent, the consequences of earlier emergence and activity for the tick population dynamics and disease risk are not well understood [115]. Most models that incorporate these temperature-dependent developmental rates have assumed constant mortality rates during development and questing [53]. Thus, longer development and activity periods inevitably lead to reduced survival. While recent models by Dobson *et al.* [30,87] and Estrada-Pena *et al.* [31] incorporate more realistic controls on questing behaviour and, in the former, host contact rates, these are still founded on best guesses and scant relationships derived from little empirical evidence. Experimental studies of the effects of condition-dependent tick behaviour on population dynamics and host–vector–pathogen encounter rates are needed.

There is a need for theory and empirical studies of tick questing behaviour and particularly success in finding a host under various climatic conditions. To what degree are questing ticks limited by conditions (i.e. by time available to quest) versus by host density? Of course these are not independent—if hosts are abundant then even a small amount of time questing may suffice—but the precise relationship between these two aspects of host-finding is not clear, and may be critical [98]. If, for instance, host density is more important than climatic constraints on questing success over a range of conditions, this would change the focus of discussion. Note, too, that the discussion so far assumes that host density and activity are not influenced by climatic conditions, but neither assumption is safe. In particular, it will be important to determine how host populations may change in distribution and abundance in a changing climate.

Given the long and complex life cycle of *Ixodes* species, it is not surprising that ticks in different regions appear to be constrained by different climatic influences [32]. Focusing on the relative influence of climate on development, questing and host encounters, and overwintering survival in different regions may prove fruitful. Models that embody alternative hypotheses also need to be compared in a robust statistical framework.

There is also a need to better understand what controls the phenology of nymphal emergence and activity. (At least in North America, it appears that larval emergence is controlled by temperature-dependent rates of development.) If the emergence of nymphs is not strongly influenced by changing temperatures or has a different relationship with temperature than larvae, then there is potential for increasing or decreasing delays between larval and nymphal activity and therefore for selection favouring different strains of pathogens.

For mosquito-borne diseases such as malaria and dengue fever, experimental and modelling studies have demonstrated that climate variability in the form of daily temperature range can affect risk of disease transmission [118,119]. In both cases, models suggest that incorporating daily temperature variation (as opposed to including only a daily mean temperature) when baseline conditions are cool would increase the probability of an outbreak. However, when baseline conditions are hot, including a daily temperature range is likely to reduce the chance of an outbreak. To date, studies of the effects of temperature variability on the dynamics of tick-borne disease appear to be lacking, but variability could be important nevertheless [58,120]. Climate variability could affect tick survival, host-seeking patterns and phenology, as well as replication rates of tick-borne pathogens.

Another major challenge in understanding the relative importance of directional climate change in dynamics of tick-borne disease is disentangling the climatic effects from temporal variability unrelated to climate change. Recent studies in other disease systems, such as cholera and malaria, have provided models in which causes of fluctuating disease risk from factors intrinsic to the parasites and hosts are distinguished from extrinsic factors such as warming trends [83,121]. Similar approaches applied to long-term datasets on tick-borne disease could be illuminating. Data–model fusion approaches using hierarchical state–space structures also can be useful in integrating multiple data sources (e.g. entomological and epidemiological), spatial scales, and explicitly incorporate uncertainty in observations and latent processes [122]. Such approaches have rarely been applied to tick-borne diseases under changing climate, but would greatly facilitate the objective comparison of alternative models (i.e. hypotheses) in a coherent framework.

To date, there has been little consideration of how environmental conditions such as temperature or temperature variation might influence the replication and persistence of pathogens (e.g. *B. burgdorferi*) in the ticks themselves. Several studies have shown that replication rates of mosquito-borne pathogens increase with temperature (e.g. [123]), but we are aware of no studies addressing this issue for tick-borne pathogens. Alternatively, the immune responses of many invertebrates are temperature sensitive, so it is possible that ticks will be more likely to reduce pathogen densities or even clear their infections at warmer temperatures. In either case, environmental temperatures after the first blood meal have the potential to alter the effective transmission rate from hosts to ticks.

Lastly, efforts to predict the distribution and risk of tick-borne disease necessarily assume that whatever mechanisms control ticks and tick-borne disease currently will be at work in the future. In other words, there is an implicit assumption that ticks, their hosts and the pathogens they carry are not evolving rapidly relative to climate change. This assumption warrants further attention (e.g. [124]).
